# Management of an incidentally discovered pancreatic neoplasm mimicking an ovarian neoplasm during pregnancy: A case report

**DOI:** 10.1097/MD.0000000000051361

**Published:** 2026-10-30

**Authors:** Min Jung Baek, Min Jeong Kwak, Haeyoun Kang, Ji Yoon Kim, Ji Hyon Jang, Eun Duc Na

**Affiliations:** a Department of Obstetrics and Gynecology, CHA Bundang Medical Center, CHA University, Seongnam, Korea; b Department of Pathology, CHA Bundang Medical Center, CHA University, Seongnam, Korea.

**Keywords:** case report, high grade dysplasia, pancreatic mucinous cystic neoplasm, pregnancy, surgical timing

## Abstract

**Rationale:**

Mucinous cystic neoplasms (MCNs) of the pancreas are uncommon tumors that occur during pregnancy, but because MCNs of the pancreas are sex hormone responsive, these tumors can grow rapidly. We report a case of MCN of the pancreas that was diagnosed during pregnancy.

**Patient concerns:**

A 19.4 × 14.5 cm lesion was detected in the left upper abdomen in a 36-year-old asymptomatic woman with chronic hypertension and diet-controlled gestational diabetes at a gestational age (GA) of 36 weeks.

**Diagnoses:**

Magnetic resonance imaging showed a multiseptated cystic mass with a regular thin wall arising from the pancreatic tail, and final pathological findings identified a pancreatic MCN with high-grade neoplasia and no metastasis.

**Interventions:**

At a GA of 37^+2^ weeks, acute severe hypertension raised suspicion for superimposed preeclampsia, and cesarean delivery was performed. Laparoscopic distal pancreatectomy with splenectomy was performed 11 weeks postpartum.

**Outcomes:**

The postoperative course was uncomplicated, and a 6-month follow-up was planned without further treatment.

**Lessons:**

The optimal timing of surgical resection for MCNs of the pancreas in pregnancy should be determined by the GA and the potential for malignancy. If malignancy is not suspected, planned postpartum resection is a safe approach.

## 1. Introduction

Mucinous cystic neoplasms (MCN) of the pancreas are uncommon tumors that account for 2% of all pancreatic tumors^[1]^ and comprise up to 25% of all resected cystic neoplasms of the pancreas.^[2]^ MCNs are usually identified in the body or tail of the pancreas, almost exclusively observed in females, and are sex hormone responsive. An ovarian-like stroma under the epithelium is a characteristic histopathological feature of MCN. Thus, pancreatic MCN stromal cells have been shown to be positive for estrogen and progesterone receptors. Thus, it can grow rapidly during pregnancy despite being an extremely rare disease in pregnancy.^[3]^

We report a case where MCN was diagnosed during pregnancy and present a review of the available literature about MCN during pregnancy.

## 2. Case presentation

A 36-year-old nulliparous, gravida 1, woman had undergone antenatal care in our institution since the early stages of pregnancy. She first visited our institution after confirmation of a self-administered urinary human chorionic gonadotropin (hCG) test as positive. At that time her body weight was 72 kg and her body mass index (BMI) was 26.4 kg/m^2^. She was diagnosed with hypertension at a gestational age (GA) of 5 weeks at her first hospital visit and was prescribed anti-hypertensive medication. Furthermore, she was diagnosed with gestational diabetes at a GA of 27 weeks and was treated with diet control and exercise. Otherwise she did not experience any significant maternal or fetal associated problems until a GA of 36 weeks.

At a GA of 36 weeks, a huge cystic lesion (19.4 × 14.5 cm) with a well-defined margin was incidentally detected in the left upper abdomen with routine obstetric sonography (Fig. 1). In spite of this large volumetric lesion, she had not experienced any discomfort. Non-contrast Magnetic resonance imaging (MRI) at a GA of 36^+4^ weeks revealed a multi-septated cystic mass with a regular thin wall, originating from the tail of the pancreas (Fig. 2). After an extensive consultation with the patient, obstetric doctors, and general surgeons, the patient decided to attempt a vaginal delivery and then undergo a laparoscopic operation for excision of the tumor.

**Figure 1.**
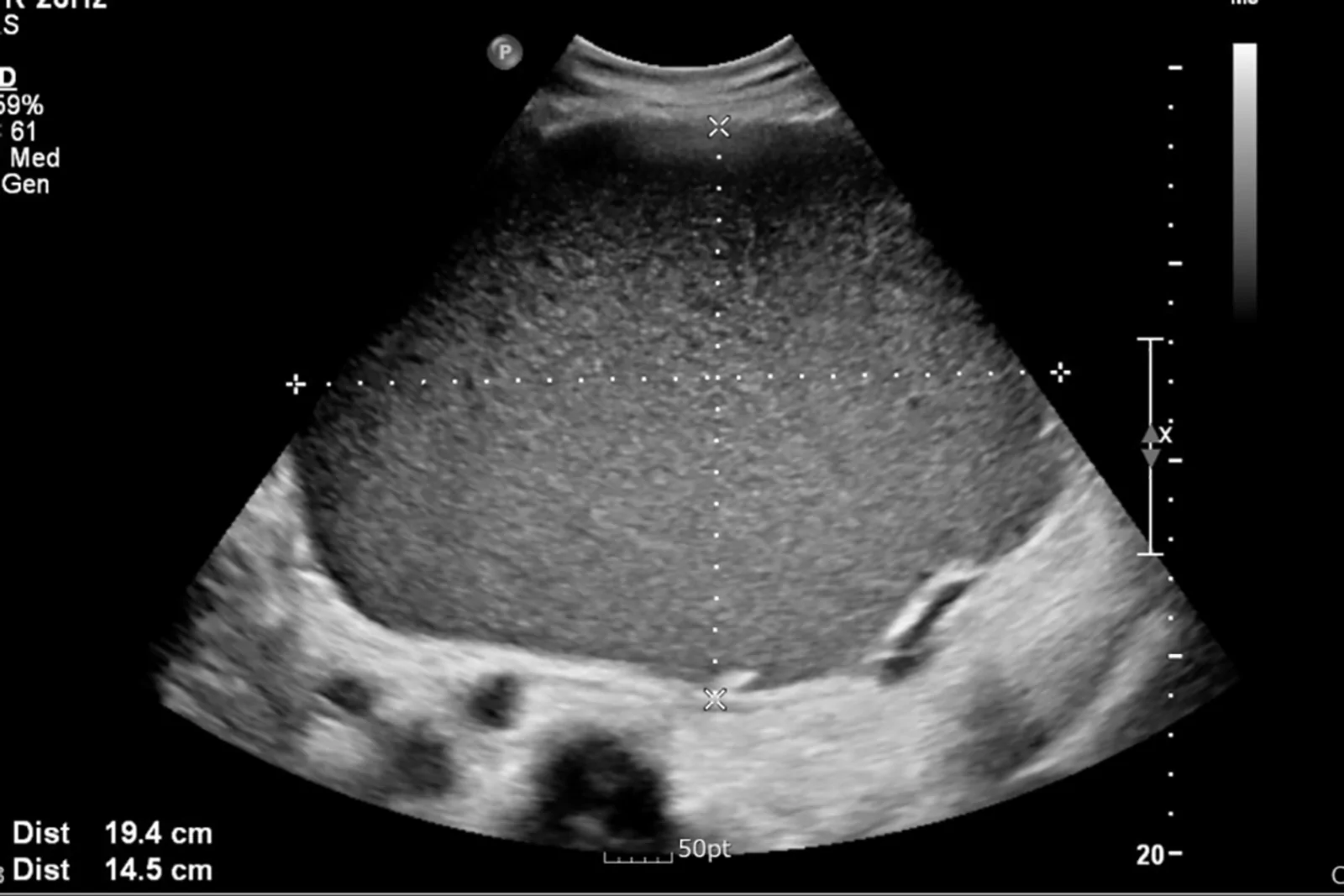
Unilocular large cystic lesion (19.4 × 14.5cm) with a ground glass echoic pattern.

**Figure 2.**
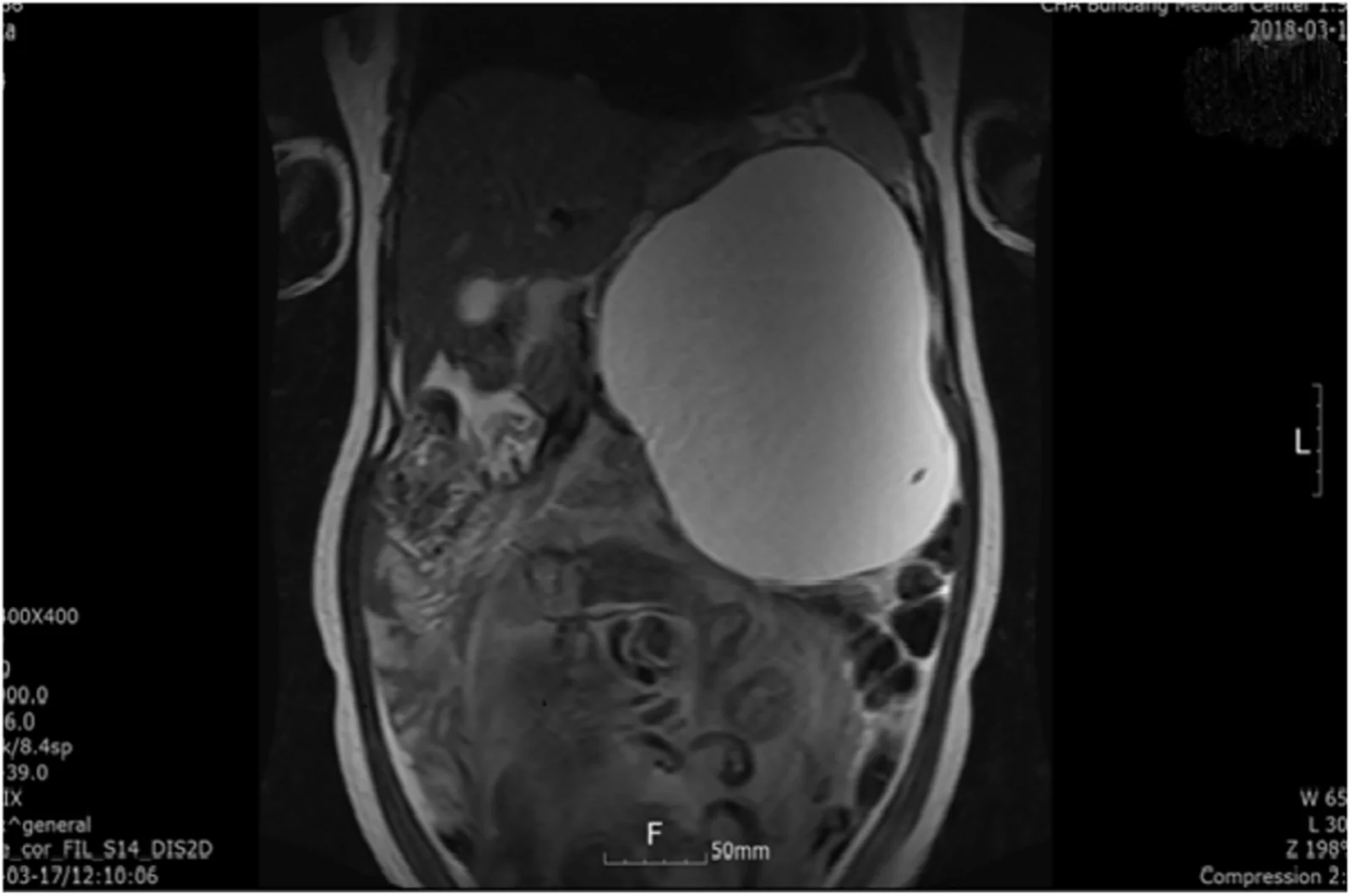
A coronal view of the cystic neoplasm observed with magnetic resonance imaging (MRI). MRI = magnetic resonance imaging.

However, at GA 37^+2^ weeks, she presented to the emergency room with a newly onset severe headache. Her blood pressure acutely escalated to 170/120 mm Hg. Initial laboratory investigations revealed no proteinuria, and her platelet count, liver function, and serum creatinine levels were normal. The development of new-onset, persistent neurological symptoms such as a severe headache in a patient with preexisting chronic hypertension constitutes a definite diagnosis of superimposed preeclampsia with severe features, even in the complete absence of proteinuria or other laboratory abnormalities. Given the uncontrolled severe hypertension despite anti-hypertensive medication, the imminent risk of maternal cerebrovascular complications or placenta abruption, and an unripe uterine cervix (Bishop score < 4) that predicted a high risk of failed induction, an immediate intervention was mandated. Therefore, a cesarean delivery was subsequently performed as the most appropriate and justified mode of delivery. The procedure was uncomplicated. The newborn baby was male, weighed 3.07 kg, and the Apgar score was 8 and 9 at 1 and 5 minutes, respectively. The immediate maternal and neonatal postpartum courses were uncomplicated.

Pancreatic surgery was not performed concurrently with cesarean delivery. In the absence of imaging findings strongly suggestive of invasive disease other than the very large tumor size, the multidisciplinary team favored a staged approach to avoid adding a major pancreatic resection and splenectomy to an obstetric procedure performed for a maternal hypertensive indication, thereby limiting prolonged anesthesia, operative blood loss, postoperative morbidity, and the complexity of immediate postpartum recovery. Serum CEA and CA 19-9 were not measured during the postpartum preoperative period. On the day before pancreatic surgery, serum amylase was 64 U/L, lipase 71 U/L, and total bilirubin 0.88 mg/dL; AST and ALT were within the normal range. No contrast-enhanced CT was obtained before pancreatic resection after delivery. Because the lesion had already been localized and characterized on antenatal MRI and definitive resection was planned, the surgical team proceeded to surgery based on the MRI findings and clinical assessment.

Eleven weeks after delivery, laparoscopic distal pancreatectomy with splenectomy was performed. Grossly, a well-defined large unilocular tumor, 20 cm in diameter with a thick fibrous wall, was noted in the distal pancreas. This tumor contained 3 liters of brownish infected fluid and extended over the distal pancreas and spleen, but it did not involve the adrenal gland. The tumor was completely removed, and the margins of the specimen and spleen were free of disease. It weighed 555 g in total; the external surface of the tumor was smooth, and the inner surface showed partial septations and trabeculations with a whitish-gray and partly yellowish appearance; no solid or papillary lesions were noted. There was no evidence of connection of the cyst to the pancreatic duct. Microscopically, the cyst was lined with a columnar mucinous epithelium of intestinal-like morphology with multifocal low-grade and high-grade dysplasia. It showed an ovarian-like stroma typical of MCN of the pancreas (Fig. 3). The pathologic diagnosis was MCN with a high grade of dysplasia. The immunohistochemical stain was positive for estrogen receptor (ER; Fig. 4A) and Ki-67 (Fig. 4B). Additionally, the tumor cells were positive for p53. Moreover, there were 5 enlarged peripancreatic lymph nodes; however, these did not show any evidence of metastasis, just reactive hyperplasia. The clinical stage was classified as T_IS_N_0_M_0._

**Figure 3.**
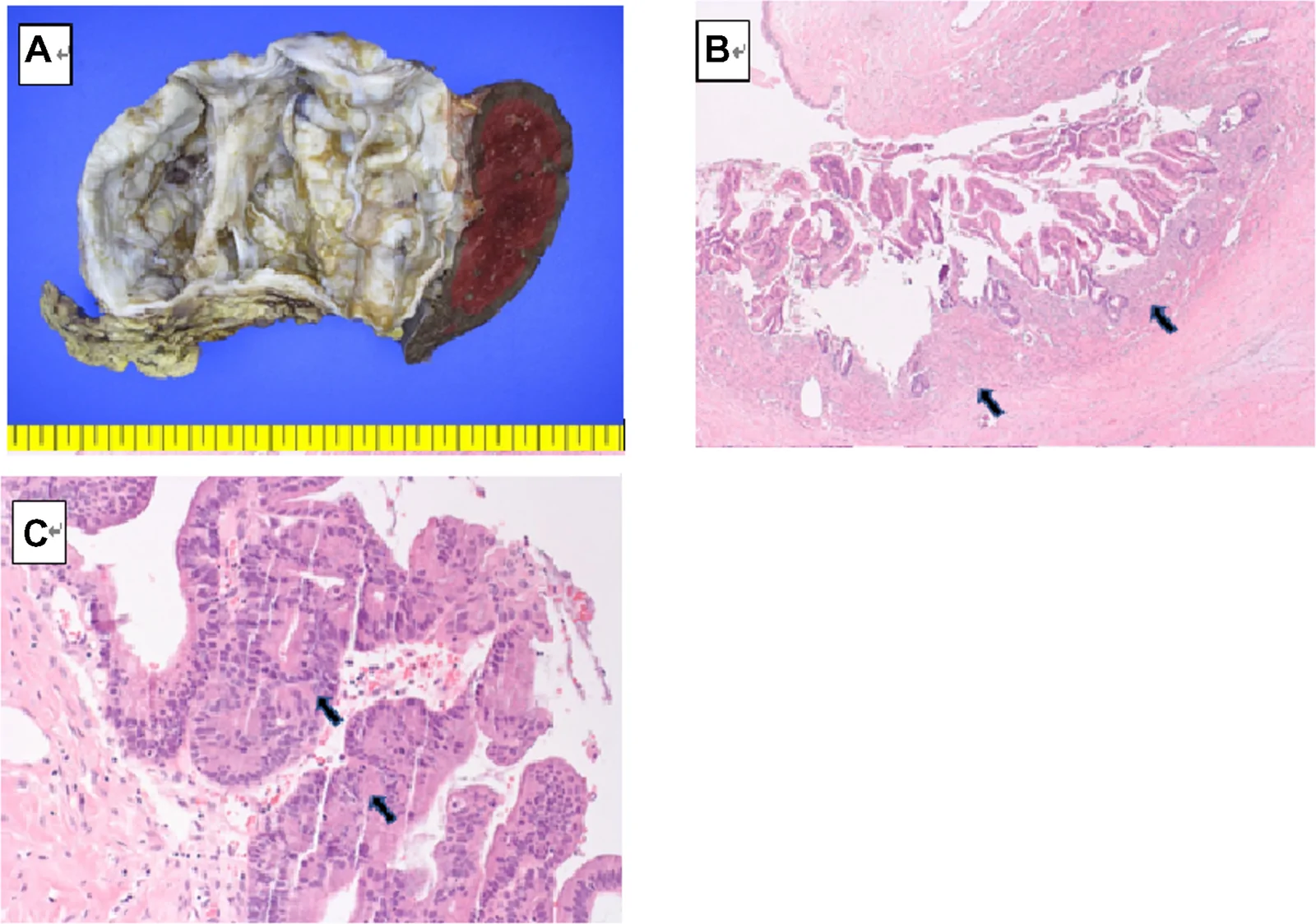
(A) Grossly, the distal pancreatic mass was a well-defined unilocular cyst with a fibrotic wall. The inner surface showed partial septations and trabeculations with a whitish gray and partly yellowish appearance. The mass had adhesions with the spleen. (B) A high-power view of the cyst with observable dysplastic epithelium and ovarian-like stroma. The arrows indicate the ovarian-like stroma. (H&E, ×40) (C) The arrows indicate the area with a high grade of dysplasia. Frequent mitosis, a high nuclear to cytoplasmic ratio, and a loss of orientation were observed in the tissue (H&E, ×200).

**Figure 4.**
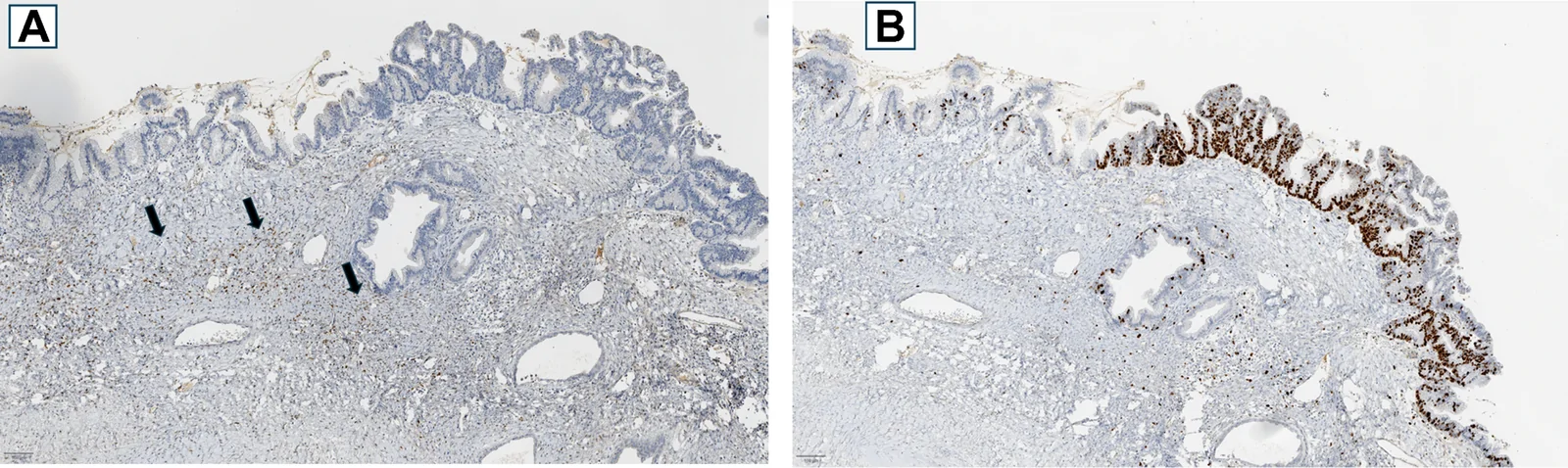
Immunohistochemical staining of the pancreatic mucinous cystic neoplasm. (A) Ovarian-like stromal cells indicated by the arrow show strong nuclear positivity for estrogen receptor (ER, ×8). (B) The high-grade dysplastic area of mucinous neoplasm shows an increased proliferative index (right) compared to the adjacent low-grade area (left; Ki-67, ×8).

One week after pancreatic surgery, total bilirubin was 0.18 mg/dL, serum amylase 67 U/L, and lipase 82 U/L; AST and ALT remained within the normal range. Contrast-enhanced CT was performed after surgery as part of the postoperative evaluation. Because the tumor was noninvasive and completely resected with negative margins and no nodal metastasis, no adjuvant treatment was indicated. Follow-up was planned according to the institutional postoperative strategy.

## 3. Discussion

Pancreatic cystic neoplasm (PCN) can be divided into 4 subtypes, including MCN, intraductal papillary mucinous cystic neoplasm (IPMN), serous cystadenomas, and solid pseudopapillary neoplasm. MCNs typically arise in females and usually occur in the pancreatic body or tail. Because of the presence of a mucin-producing epithelial lining, MCNs are histologically similar to IPMNs. However, the “ovarian-like stroma” observed in MCNs, and the fact that MCNs do not interact with the pancreatic ductal system can distinguish it from IPMNs.^[4,5]^

All MCNs have a risk of malignant transformation; hence, resection of the tumor is generally the first line treatment in MCN patients. The majority of MCNs can be classified into mucinous cystadenoma, mucinous cystic tumor, borderline, and mucinous cystadenocarcinoma. At the time of resection, the prevalence of invasive carcinomas in MCNs ranges from 6% to 36%.^[6]^ The factors for predicting the malignancy of MCNs are large tumor size, mural nodules, and thickening of the septum.^[7]^ In our patient, although the tumor was extraordinarily large, it lacked other suspicious findings such as solid components or septal thickening. This favorable morphology allowed us to consider extending the timing for surgical resection until after delivery.

Pancreatic neoplasms are rare during pregnancy, with <25 reported cases.^[8,9]^ The most common pancreatic neoplasms in pregnancy are MCN and adenocarcinomas_._^[10]^ Diagnosing an MCN during gestation is exceptionally challenging, particularly because early-stage lesions are often entirely asymptomatic. Furthermore, even when pregnant women present with symptoms such as upper abdominal discomfort, dyspepsia, and shortness of breath, it is not easy to differentiate these from normal occurrences in pregnancy.

Furthermore, as the pregnancy advances into the third trimester, the rapidly growing gravid uterus drastically alters the intra-abdominal anatomy and displaces surrounding organs. Consequently, a large upper abdominal cystic lesion can be easily misidentified or mistaken for an ovarian/adnexal mass, a degenerating uterine leiomyoma, or alternative retroperitoneal and mesenteric cysts. This anatomical crowding emphasizes the critical necessity of careful, high-resolution cross-sectional imaging to definitively establish the organ of origin. In our patient, while routine obstetric ultrasonography initially detected the massive lesion incidentally, subsequent magnetic resonance imaging (MRI) was indispensable in localizing the multi-septated, thin-walled cystic mass specifically to the pancreatic tail, thereby preventing a potential misdiagnosis.

To our knowledge, there have been 20 reported cases of pancreatic MCN detected during pregnancy or shortly after delivery since the first case was reported in 1986.^[11]^ Twelve cases were operated on during pregnancy, and the rest of the cases were operated on after delivery. Regarding the histopathological results, 6 cases were diagnosed with adenocarcinoma, and 14 cases were diagnosed with benign MCN. The pathologic results in 3 out of 8 cases operated on after delivery were adenocarcinoma, and in 3 out of 12 cases operated on during pregnancy were adenocarcinoma. Notably, no miscarriages or operation-related maternal deaths were reported, demonstrating that surgical intervention can be safely performed when indicated.

MCNs are observed almost exclusively in women. The responsiveness of the tumor to sex hormones may contribute to its occurrence during pregnancy; however, its association with pregnancy is unclear. Cystic tumors in the pancreas grow slowly and are indolent for several years. But MCNs of the pancreas that develop during pregnancy can be large, and studies have reported that MCNs increase in size in a short time during pregnancy.^[12,13]^ Therefore, fetal growth restriction (FGR) can occur; however, in our case, there was no observed FGR. Furthermore, ruptures of the MCN during pregnancy have been reported in some cases.^[14]^ During pregnancy, although PCN rupture is extremely rare, when acute abdominal pain is experienced during pregnancy, rupture of the PCN should be suspected. In addition to these case reports, a recent multicenter case control study by Fogliati et al (2024) analyzed a larger cohort of resected MCNs, noting that peri-gestational MCNs are significantly associated with an increased cyst diameter and carry a 29% rate of high-grade dysplasia of invasive carcinoma, which further underscores the clinical significance of these tumors during gestation.^[15]^

The timing of pancreatic resection during pregnancy must balance oncologic urgency against maternal, fetal, anesthetic, and technical risks. Surgery during pregnancy may be justified when imaging suggests invasive malignancy, rapid progression, rupture, hemorrhage, severe symptoms, or other complications that make postponement unsafe. Conversely, when a lesion is detected late in gestation and lacks clear evidence of invasive disease, delivery followed by postpartum pancreatic surgery can be a reasonable staged strategy. In our case, detection at 36 weeks, predominantly cystic thin-walled morphology, and absence of tumor-related symptoms supported postponement of pancreatectomy until after delivery. Delivery then became necessary for a separate obstetric indication: severe-range chronic hypertension accompanied by a severe headache despite treatment. A simultaneous distal pancreatectomy and splenectomy at cesarean delivery was avoided because it would have combined an obstetric operation with major upper abdominal surgery, increasing anesthetic duration, bleeding risk, postoperative complexity, and the burden of recovery in the immediate postpartum period.

However, a staged approach requires appropriate reassessment rather than simple delay. Maternal symptoms, interval tumor growth and morphology, pancreatic and hepatobiliary laboratory parameters, tumor markers, and cross-sectional imaging may be considered according to the clinical situation. In this patient, serum CEA and CA 19-9 were not measured either during pregnancy or before pancreatic resection; therefore, tumor-marker-based biochemical risk stratification was not available. On the day before surgery, serum amylase was 64 U/L, lipase 71 U/L, and total bilirubin 0.88 mg/dL, and AST and ALT were within the normal range. Serum tumor markers are adjunctive rather than definitive tests and cannot by themselves establish or exclude high-grade dysplasia or invasive carcinoma; nevertheless, the absence of CEA and CA 19-9 measurements is a limitation of this case. No contrast-enhanced CT was obtained during the postpartum preoperative interval. The surgical team proceeded to definitive resection using the antenatal MRI and clinical assessment, and contrast-enhanced CT was subsequently obtained after surgery. We acknowledge that a preoperative contrast-enhanced CT could have provided additional assessment of local extension, regional lymph nodes, and distant metastatic disease before resection. Thus, the absence of a complete preoperative CT-based staging evaluation is an additional limitation of this case.

In summary, this report has important limitations. It describes a single patient with a rare pregnancy-associated pancreatic tumor, and conclusions about the safest timing of resection cannot be generalized. In addition, the lesion was first recognized late in gestation, so its growth trajectory during most of pregnancy was unknown, and serial tumor-size measurements during the postpartum interval were not available for this report. Serum CEA and CA 19-9 were not measured, limiting biochemical risk stratification, and no contrast-enhanced CT-based staging evaluation was performed before pancreatic resection. Although postoperative contrast-enhanced CT was obtained, it does not substitute for the potential staging information that could have been available before surgery. Nevertheless, this case highlights practical issues that recur in published reports: establishing the pancreatic origin of a large abdominal cyst, estimating malignant potential with pregnancy-compatible imaging, separating obstetric indications for delivery from oncologic indications for resection, and using multidisciplinary planning when surgery can be safely staged.

## 4. Conclusion

Pancreatic MCN should be considered when a large upper abdominal or apparently adnexal cystic mass is detected during pregnancy. Features that increase concern for high-grade dysplasia or invasive disease include large size, mural nodules or solid components, wall or septal thickening, suspicious lymphadenopathy, rapid growth, symptoms, and, when available, elevated serum CA 19-9. Serum tumor markers are adjunctive and should be interpreted together with clinical and radiological findings. When surgery is deferred, interval clinical reassessment and appropriate staging before definitive resection are important, and the management plan should be reevaluated if concerning clinical, biochemical, or radiologic changes emerge. The timing of imaging, delivery, and pancreatic resection should be individualized through multidisciplinary consultation according to gestational age, maternal-fetal condition, and the estimated malignant potential of the lesion.

## Author contributions

**Conceptualization:** Eun Duc Na.

**Data curation:** Min Jeong Kwak, Ji Yoon Kim, Ji Hyon Jang.

**Formal analysis:** Haeyoun Kang.

**Investigation:** Haeyoun Kang, Ji Yoon Kim.

**Project administration:** Haeyoun Kang, Eun Duc Na.

**Resources:** Min Jeong Kwak.

**Supervision:** Eun Duc Na.

**Visualization:** Haeyoun Kang.

**Writing – review & editing:** Min Jeong Kwak, Eun Duc Na.

**Writing – original draft:** Min Jung Baek, Eun Duc Na.
